# Supplementary material for: Latent Dirichlet allocation mixture models for nucleotide sequence analysis
Source: NAR Genom Bioinform. 2024 Aug 9;6(3):lqae099. doi: 10.1093/nargab/lqae099 (PMC11310860; doi:10.1093/nargab/lqae099)
Supplement: lqae099_Supplemental_File [file lqae099_supplemental_file.docx]

**Latent Dirichlet Allocation Mixture Models for Nucleotide Sequence Analysis**

Bixuan Wang and Stephen M. Mount*

Dept. of Cell Biology and Molecular Genetics, University of Maryland, College Park, MD 20742

*: Corresponding author

Stephen M. Mount

smount@umd.edu

## **Supplemental Figures and Legends**


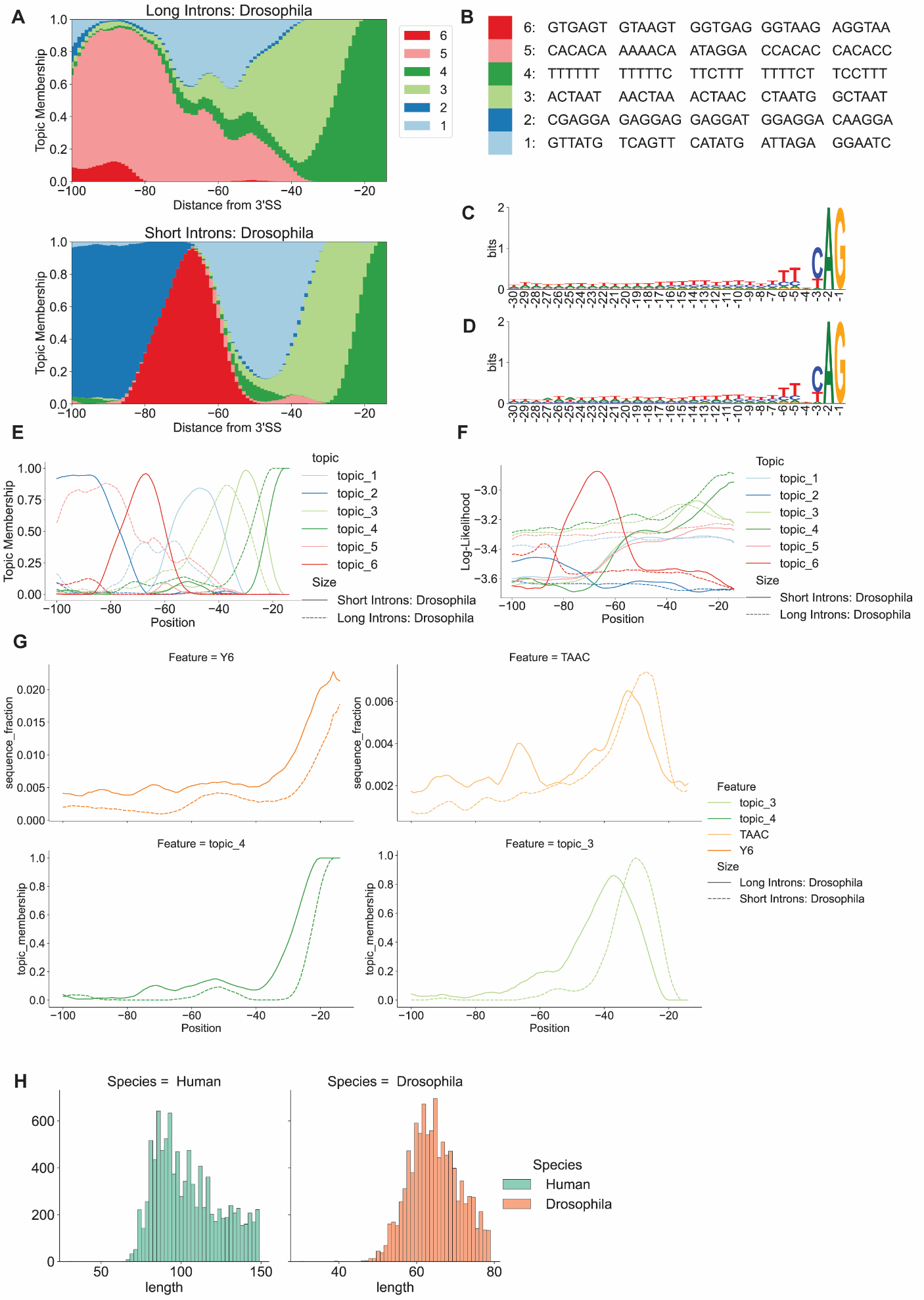


**Figure S1. LDA characterization of short and long Drosophila introns using aligned positions as samples.**

LDA was applied to samples corresponding to positions in an alignment of 3’ splice sites from

long and short introns from Drosophila (long>=80 nt.; short<80 nt.).

A. Structure plots of long (n=10,000) and short (n=10,000) Drosophila introns. Hexamer features starting at positions -100 to -13 upstream of 3’ splice sites (encompassing -100 to -3) were used as samples.

B. Table of top 5 enriched hexamer features in the six topics in D.

C. Sequence logo of 3’SS of long Drosophila introns.

D. Sequence logo of 3’SS of short Drosophila introns.

E. Line plot of topic distribution across positions relative to the 3’SS of Drosophila introns.

F. Line plot of the likelihood of observing the distribution of features at each position in Drosophila introns.

G. Line plots of the branch site and pyrimidine tract consensus motifs and corresponding topic signals.

H. The distribution of short intron sizes in the analysis of Fig 2 and Fig S1.


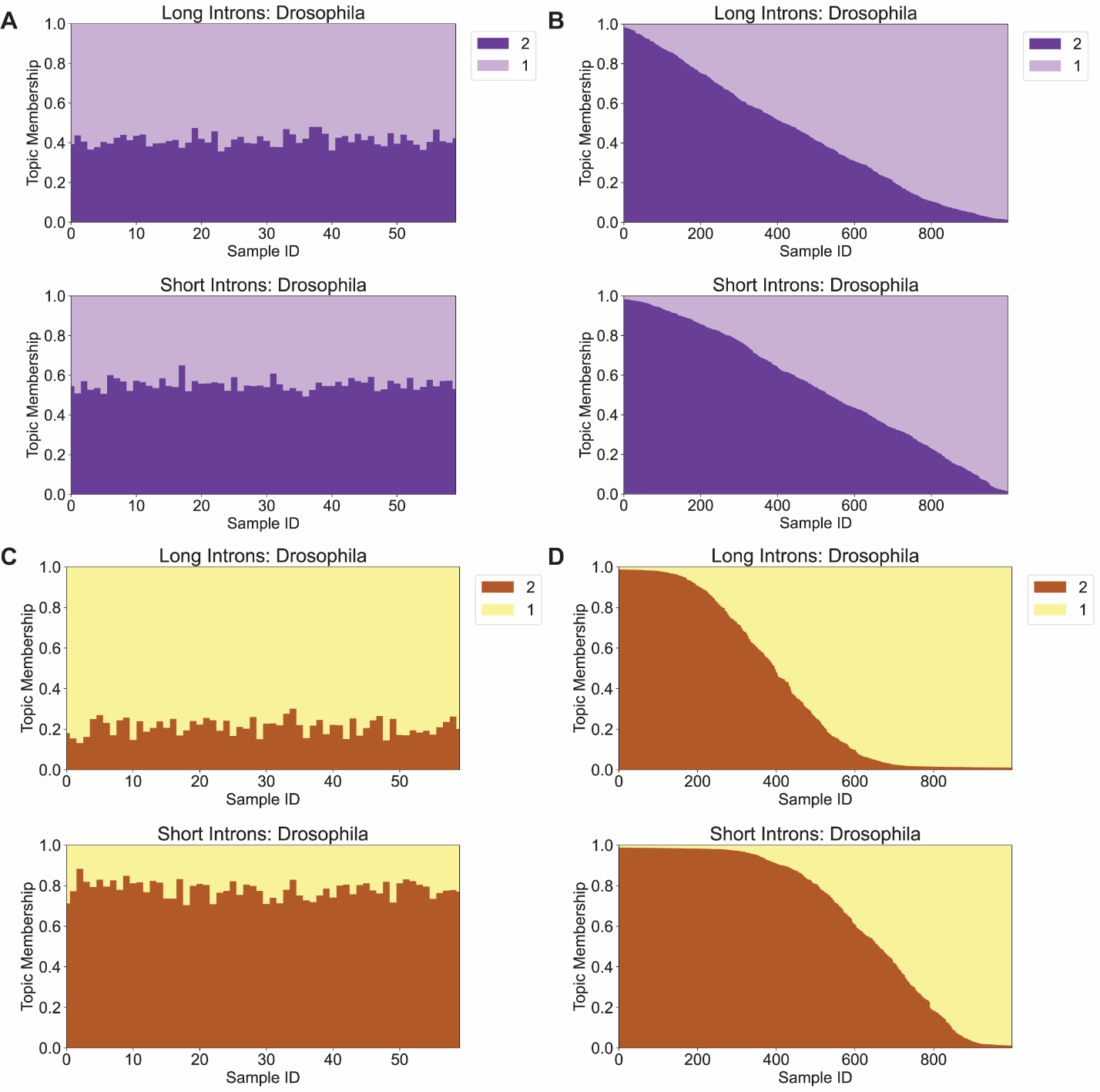


**Figure S2. Non-positional feature analysis can not distinguish intron subtypes from Drosophila introns.**

LDA was applied to Drosophila intron sequences to identify differences between long and short introns using single sequences. Unlike the result in Fig S1, non-positional features are not able to describe the differences between the two groups.

A. Structure plots of long (n=15,000) and short (n=15,000) Drosophila intron sequences (30 nt. of exon and 50 nt. of intron) near 3’SS. Every sample contains tetramer features from 250 sequences (15,000 sequences total).

B. Structure plots of long (n=2,000) and short (n=2,000) Drosophila intron sequences near 3’SS. Every sample is a single sequence. The topics are the same as the topics in Fig S2A.

C. Structure plots of long (n=15,000) and short (n=15,000) Drosophila intron sequences (30 nt. Of exon and 50 nt. Of intron) near 5’SS. Every sample contains tetramer features from 250 sequences (15,000 sequences total).

D. Structure plots of long (n=2,000) and short (n=2,000) Drosophila introns sequences near 5’SS. Every sample is a single sequence. The topics are the same as the topics in Fig S2C.


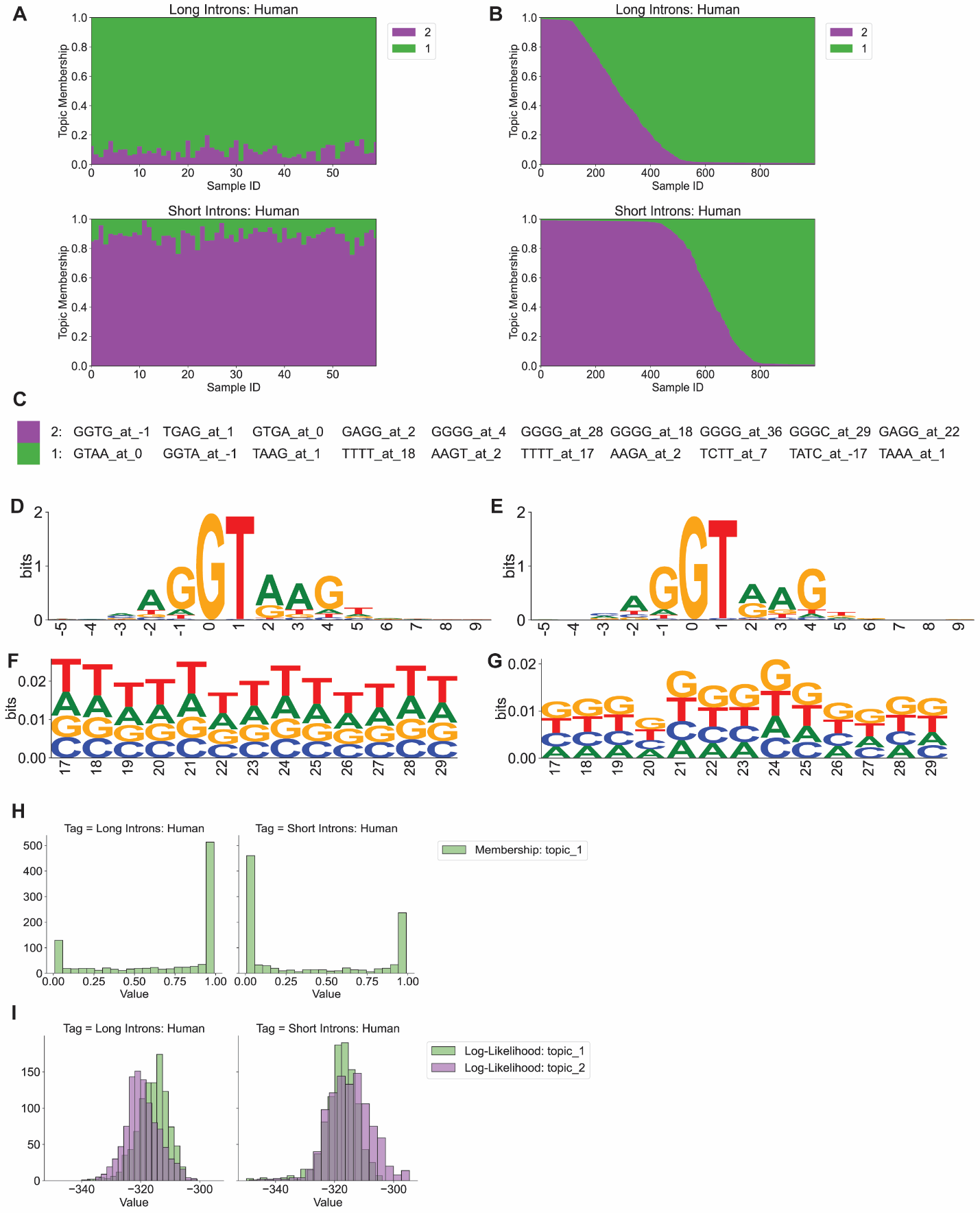


**Figure S3. LDA characterization of short and long human 5’SS using individual sequences as samples.**

LDA was applied to samples corresponding to splice site regions from long and short introns from human (long>=150 nt.; short<150 nt.).

A. Structure plots of long (n=15,000) and short (n=15,000) human intron sequences (30 nt. of exon and 50 nt. of intron) near 5’SS. Every sample contains positional tetramer features from 250 sequences (15,000 sequences total).

B. Structure plots of long (n=2,000) and short (n=2,000) human introns. Every sample is a single sequence. The topics are the same as the topics in Fig S3A.

C. Top 10 enriched positional tetramers from topics 1 and 2 from the analysis of 5’SS of human introns.

D. Sequence logo of the 5’SS from long human introns.

E. Sequence logo of the 5'SS from short human introns.

F. Sequence logo of positions 17 to 29 from long human introns.

G. Sequence logo of positions 17 to 29 from short human introns.

H. Distribution of topic 1 memberships of single sequences in Fig S3B.

I. Distribution of the likelihood of generating sequences in Fig S3B by topics.


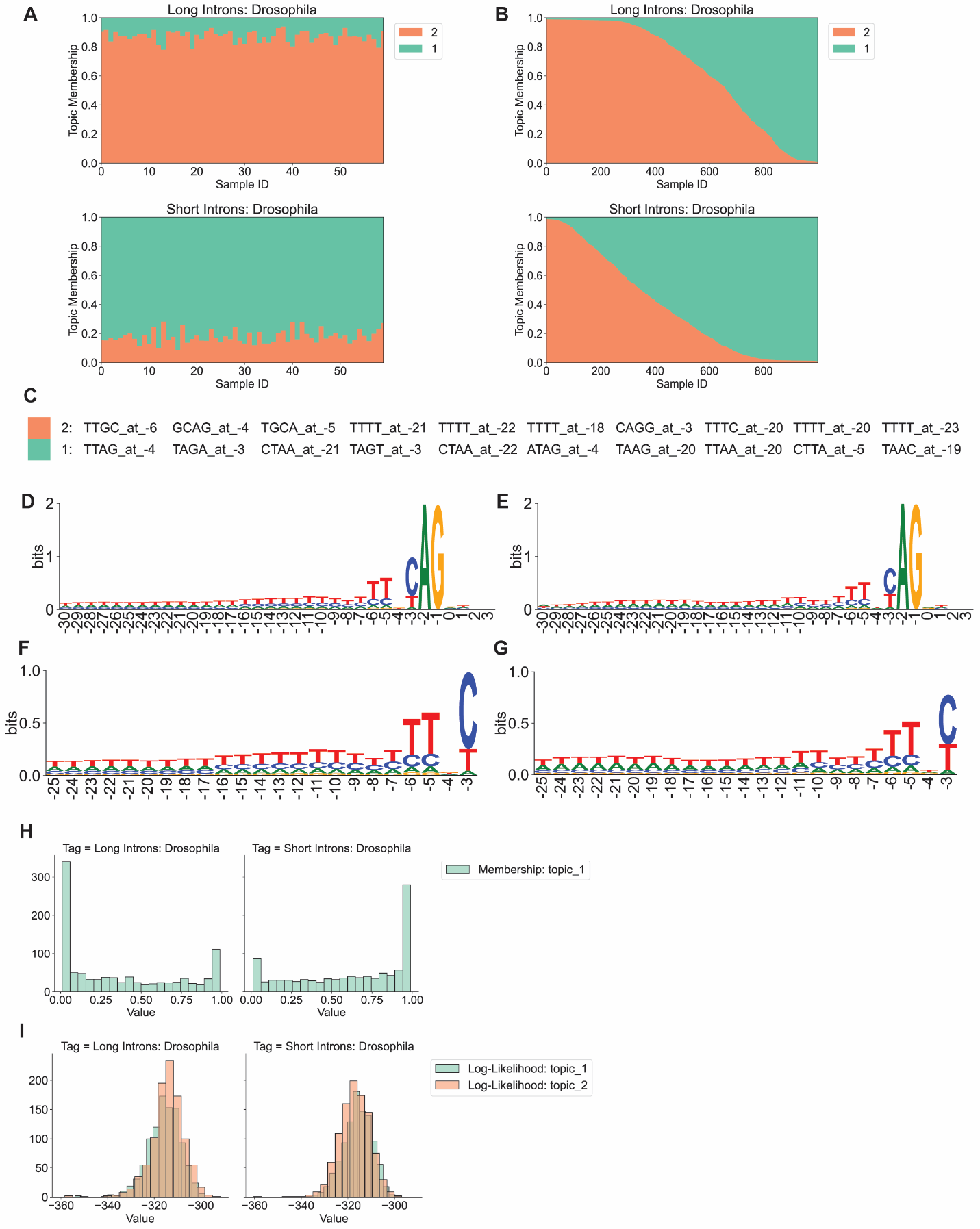


**Figure S4. LDA characterization of short and long Drosophila 3’SS using individual sequences as samples.**

LDA was applied to samples corresponding to splice site regions from long and short introns from Drosophila (long>=80 nt.; short<80 nt.).

A. Structure plots of long (n=15,000) and short (n=15,000) Drosophila intron sequences (30 nt. of exon and 50 nt. of intron) near 3’SS. Every sample contains positional tetramer features from 250 sequences (15,000 sequences total).

B. Structure plots of long (n=2,000) and short (n=2,000) Drosophila introns. Every sample is a single sequence. The topics are the same as the topics in Fig S4A.

C. Top 10 enriched positional tetramers from topics 1 and 2 from the analysis of 3’SS of Drosophila introns.

D. Sequence logo of the 3’SS from long Drosophila introns.

E. Sequence logo of the 3'SS from short Drosophila introns.

F. Sequence logo of positions -25 to -3 from long Drosophila introns.

G. Sequence logo of positions -25 to -3 from short Drosophila introns.

H. Distribution of topic 1 memberships of single sequences in Fig S4B.

I. Distribution of the likelihood of generating sequences in Fig S4B by topics.


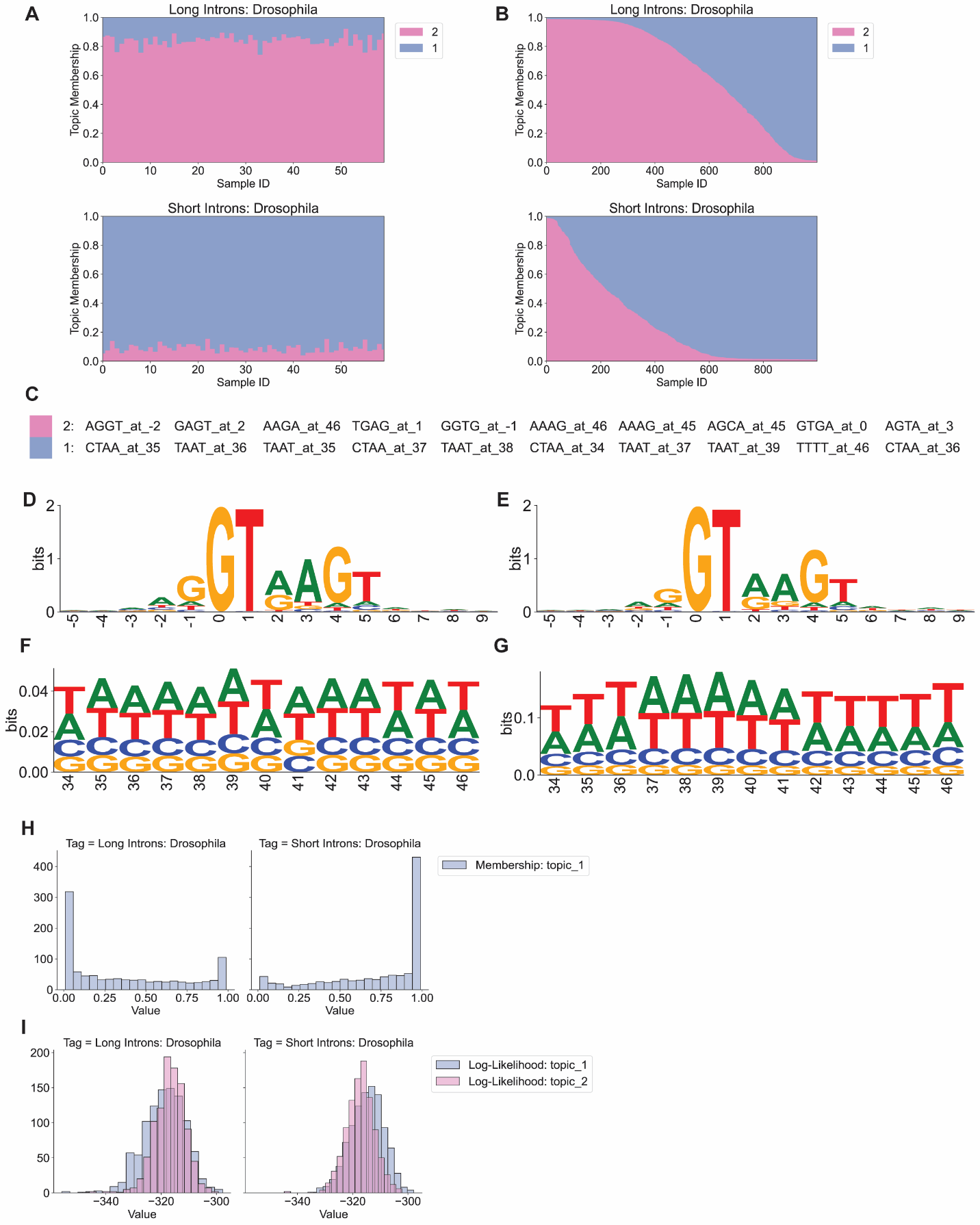


**Figure S5. LDA characterization of short and long Drosophila 5’SS using individual sequences as samples.**

LDA was applied to samples corresponding to splice site regions from long and short introns from Drosophila (long>=80 nt.; short<80 nt.).

A. Structure plots of long (n=15,000) and short (n=15,000) Drosophila intron sequences (30 nt. of exon and 50 nt. of intron) near 5’SS. Every sample contains positional tetramer features from 250 sequences (15,000 sequences total).

B. Structure plots of long (n=2,000) and short (n=2,000) Drosophila introns. Every sample is a single sequence. The topics are the same as the topics in Fig S5A.

C. Top 10 enriched positional tetramers from topics 1 and 2 from the analysis of 5’SS of Drosophila introns.

D. Sequence logo of the 5’SS from long Drosophila introns.

E. Sequence logo of the 5'SS from short Drosophila introns.

F. Sequence logo of positions 34 to 46 from long Drosophila introns.

G. Sequence logo of positions 34 to 46 from short Drosophila introns.

H. Distribution of topic 1 memberships of single sequences in Fig S5B.

I. Distribution of the likelihood of generating sequences in Fig S5B by topics.

**
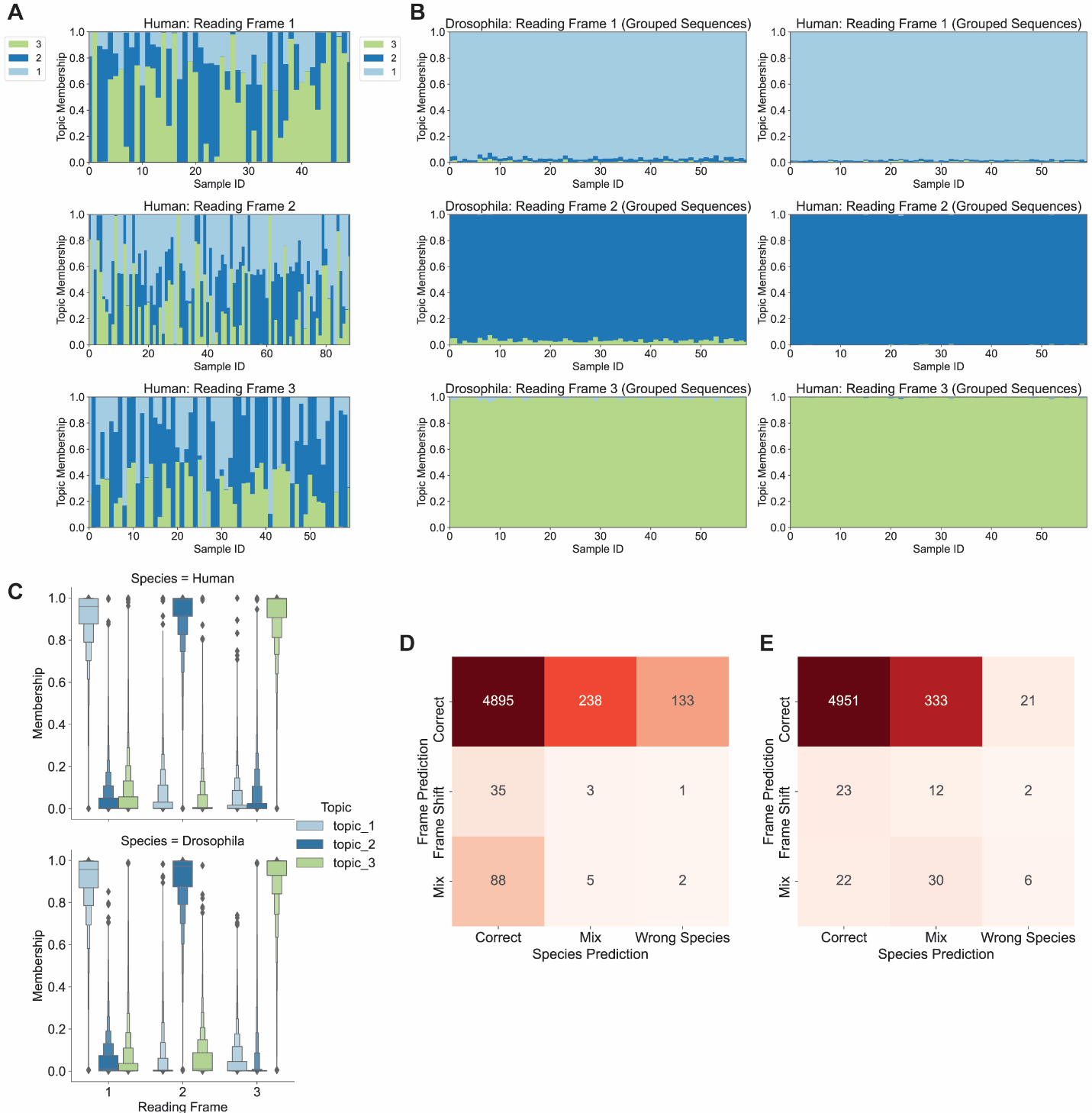
**

**Figure S6. Misclassification from LDA models and generalization of human data fitted LDA model in predicting Drosophila reading frames**.

Reading frame classification was carried out based on topic distributions. Also, the ability to distinguish reading frames by human data fitted LDA model was tested on the Drosophila sequences.

A. Topic memberships of human CDS sequences that are misclassified into incorrect reading frame tags.

B. Structure plots of transforming human and Drosophila CDS sequences with human CDS-fitted LDA model.

C. The topic distribution of sequences in Fig S6B.

D. The confusion matrix of predicting reading frames and species for human CDS using the model in Fig 6A.

E. The confusion matrix of predicting reading frames and species for Drosophila CDS using the model in Fig 6A.

**
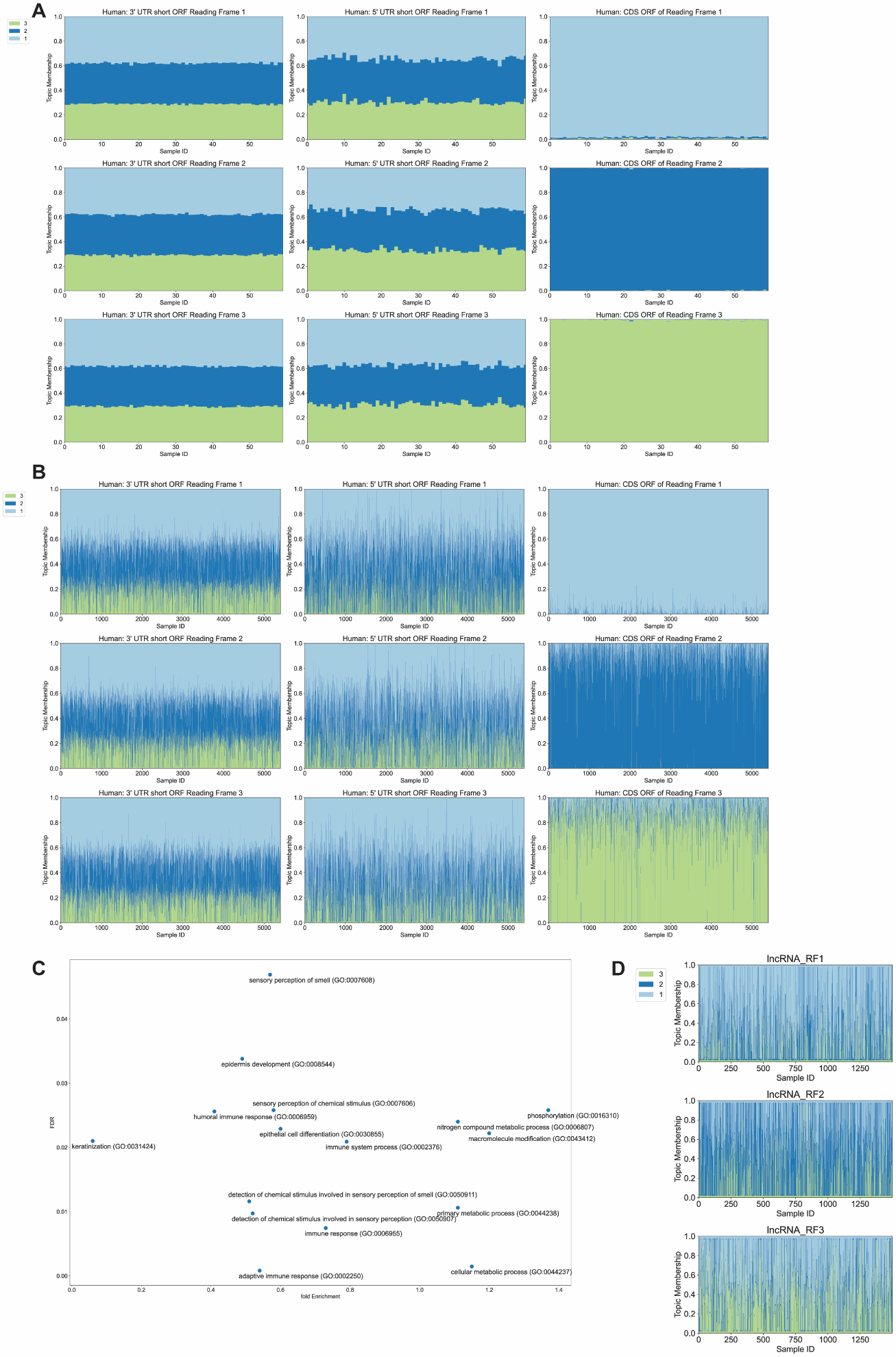
**

**Figure S7. Analyzing short ORF in human UTR5 and lncRNA by CDS LDA model.**

Human data fitted LDA model was used to analyze short ORF from non-coding sequences.

A. Structure plots of human small reading frames in UTR3, UTR5, and reading frames CDS. Data were transformed by the model Fig 4A. Each sample has hexamer compositions of 300 sequences (18,000 sequences total).

B. Structure plots of human small reading frames in UTR3, UTR5, and reading frames CDS. Each sample is a single sequence from Fig S7A.

C. Gene Ontology enrichment analysis of the smORF.

D. Structure plots of human lncRNA small reading frames. Data were transformed by the model in Fig 4A. Each sample is the hexamer composition of a single sequence (1,500 sequences are shown).


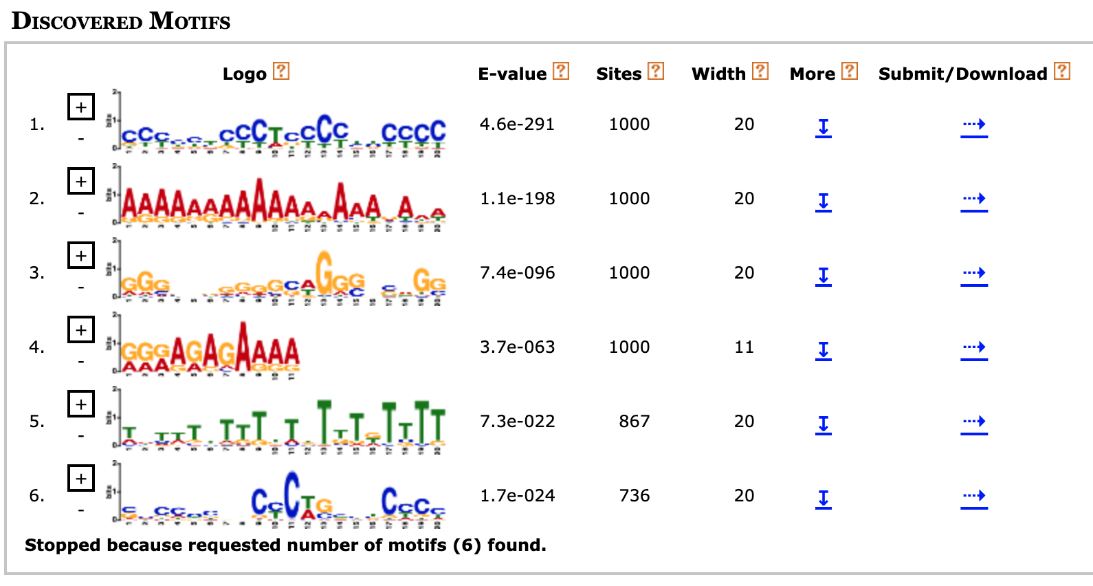


**Figure S8. MEME is unable to identify the human branch site consensus from aligned sequence in the absence of experimental data.**

The precise sequences used for identification of the human branch site motif (Fig 2) were submitted to MEME for analysis.

A. Motifs discovered by MEME using long intron sequences. The long intron sequences (Fig 2) were submitted to MEME to search for 6 motifs between 4bp and 20bp that may occur with any frequency in each sequence. Although the T-rich sequences may be the pyrimidine tract, the MEME motif did not show significant Cs in between. MEME did not discover the branch site signal either.


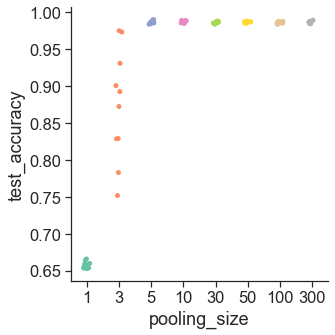


**Figure S9. Subtype identification as a function of feature counts (pooling).**

The performance of human CDS reading frame prediction based on different pooling sizes. Sequences are randomly assigned to samples, and each sample contains k-mer counts from n sequences, where n is the pooling size.

A. Distribution of test accuracies of human CDS reading frame prediction. Samples were pooled with different pooling sizes, which means the number of CDS sequences in each sample. Ten cross-validations were performed for each pooling size. 180000 CDS sequences were used in the analysis. Each cross-validation step used 14,400 sequences as the training set and 3,600 sequences as the testing set.
